# Immunologic and Protective Properties of Subunit- vs. Whole Toxoid-Derived Anti-Botulinum Equine Antitoxin

**DOI:** 10.3390/vaccines10091522

**Published:** 2022-09-14

**Authors:** Alon Ben David, Ada Barnea, Amram Torgeman, Eran Diamant, Eyal Dor, Arieh Schwartz, Osnat Rosen, Noa Caspi, Miki Saraf, Elad Lerer, Yaakov Adar, Edith Lupo, Einat Toister, Ran Zichel

**Affiliations:** 1 Department of Biotechnology, Israel Institute for Biological Research, Ness-Ziona 7410001, Israel; 2 Veterinary Center for Preclinical Research, Israel Institute for Biological Research, Ness-Ziona 7410001, Israel

**Keywords:** botulinum neurotoxin, subunit vaccine, botulinum antitoxin

## Abstract

Botulism is a paralytic disease caused by botulinum neurotoxins (BoNTs). Equine antitoxin is currently the standard therapy for botulism in human. The preparation of equine antitoxin relies on the immunization of horses with botulinum toxoid, which suffers from low yield and safety limitations. The Hc fragment of BoNTs was suggested to be a potent antibotulinum subunit vaccine. The current study presents a comparative evaluation of equine-based toxoid-derived antitoxin (TDA) and subunit-derived antitoxin (SDA). The potency of recombinant Hc/A, Hc/B, and Hc/E in mice was similar to that of toxoids of the corresponding serotypes. A single boost with Hc/E administered to a toxoid E-hyperimmune horse increased the neutralizing antibody concentration (NAC) from 250 to 850 IU/mL. Immunization of naïve horses with the recombinant subunits induced a NAC comparable to that of horses immunized with the toxoid. SDA and TDA bound common epitopes on BoNTs, as demonstrated by an in vitro competition binding assay. In vivo, SDA and TDA showed similar efficacy when administered to guinea pigs postexposure to a lethal dose of botulinum toxins. Collectively, the results of the current study suggest that recombinant BoNT subunits may replace botulinum toxoids as efficient and safe antigens for the preparation of pharmaceutical anti-botulinum equine antitoxins.

## 1. Introduction

Botulism is a neurologic and paralytic disease that is caused by botulinum neurotoxins (BoNTs) produced mainly by the bacterium *Clostridium botulinum*. BoNTs are regarded as the most potent toxins in nature and are therefore classified as tier 1 select agents by the Centers for Disease Control and Prevention (CDC) [1]. There are at least seven serotypes of BoNTs (A-G), of which serotypes A, B and E and rarely F are responsible for most cases of human botulism [2]. BoNTs are proteins of ~150 kDa consisting of a 50 kDa light chain (LC) and a 100 kDa heavy chain linked by a disulfide bridge [2].

BoNTs exert their toxicity within cholinergic nerve cells, mainly in neuromuscular junctions, by blocking exocytosis at the nerve endings, thus resulting in symmetrical cranial nerve palsies followed by descending, symmetrical flaccid paralysis. The molecular mechanism of action includes three steps [3]. First, the toxin binds to receptors on the presynaptic nerve ending. The C-terminal portion of the toxin heavy chain (designated the H_C_ fragment) is responsible for this action. Second, the LC is internalized and released into the cytosol; this process is facilitated by the translocation domain on the N-terminus of the heavy chain (H_N_). Third, one of three soluble N-ethylmaleimide-sensitive factor attachment protein receptor (SNARE) proteins is cleaved by the LC, which is a zinc-dependent endopeptidase. SNARE cleavage prevents the release of the neurotransmitter acetylcholine from nerve cells into synapses, thereby hindering the transmission of the neurologic signal to the muscle [4,5,6].

Currently, no licensed vaccine for the prevention of botulism in humans is available, and the only approved drug therapy for botulism is an immunoglobulin-based antitoxin aimed at neutralizing circulating toxin molecules. While human-derived immunoglobulin (BabyBIG^®^; Baxalta Inc., Westlake Village, CA, USA and Cangene bioPharma Inc., Baltimore, MD, USA) was developed as a specific treatment for infant botulism, the standard therapy for botulism in adults is based on treatment with equine-derived antitoxins such as BAT^®^ (Botulism Antitoxin Heptavalent, Emergent BioSolutions, Gaithersburg, MD, USA) [7] and IIBR [8]. These antibody preparations are obtained from hyperimmunized horses vaccinated with toxins or with formalin-inactivated toxins (toxoids) [5,6]. However, the toxoid manufacturing process is expensive and involves laborious efforts under strict safety conditions for its preparation. These concerns have led to the exploration of alternative approaches for the development of next-generation BoNT antitoxins. The main efforts have been focused on the establishment of a recombinant H_C_ fragment vaccine since it is nontoxic, safely produced, and carries most of the neutralizing epitopes [9,10]. Indeed, vaccination of mice with H_C_ fragment preparations elicited protective immunity against challenges with high doses of homologous toxin [11,12,13,14]. In accordance with these data, we previously reported the development of recombinant H_C_ proteins of botulinum toxins A, B, and E (H_C_/A, H_C_/B, and H_C_/E, respectively) [15,16,17].

To study the potential of H_C_ fragments to serve as alternative antigens for the preparation of botulinum antitoxin, horses were immunized in the current study with recombinant H_C_/A, H_C_/B, and H_C_/E. The immunogenicity, neutralizing potency and efficacy of these preparations are described in comparison to those of the common toxoid-derived antitoxin (TDA). The study shows that H_C_/A-, H_C_/B-, and H_C_/E-derived antitoxins present similar potency and efficacy compared to that of the TDA. This demonstrates that recombinant H_C_/A, H_C_/B, and H_C_/E can be applied instead of botulinum toxoids as effective antigens for the manufacturing of pharmaceutical anti-botulinum equine antitoxins.

## 2. Materials and Methods

### 2.1. Ethics Statement

All animal experiments were performed in accordance with Israeli law and were approved by the Ethics Committee for Animal Experiments at the Israel Institute for Biological Research (protocols: GP-02-19, approval date 17 April 2019; M-65-17, approval date 30 October 2017; and H-02-2014, approval date 11 August 2014).

### 2.2. Bacteria and Toxin

The expression and purification of H_C_/A and H_C_/B was described previously [15,16]. For H_C_/E, a synthetic gene encoding amino acids 845–1252 of BoNT/E (NCTC11219) with optimized codon usage for *E. coli* was prepared (by Genscript). The gene was cloned into pET-9a. The expression and purification of H_C_/E was as described [15]. *Clostridium botulinum* A, B, and E strains were obtained from the Israel Institute for Biological Research collection (strains A198, B592, and E450, respectively). Sequence analysis revealed conformity of the neurotoxin genes with serotypes 62A (GenBank accession number M30196, subtype A1), Danish (GenBank accession number M81186, subtype B1), and NCTC11219 (GenBank accession number X62683, subtype E1) for *Clostridium botulinum* types A, B, and E, respectively [18,19,20]. Toxins were prepared from concentrated supernatants of cultures grown for 6 days in anaerobic culture tubes. BoNT/E was activated with trypsin (0.1% at 37 °C for 45 min). The activity of all toxin preparations was at least 3 × 10^5^ mouse 50% lethal dose (MsLD_50_)/mL. BoNT toxoids were prepared by incubation of the toxin in the presence of 0.2% formalin at 30 °C for 28 days, followed by extensive dialysis against 50 mM citrate buffer (pH 5.5).

### 2.3. Potency in Mice

Mice (CD-1, *n* = 5, Charles River UK) were vaccinated with nine doses of HC fragment or toxoid (2-fold dilution factor between sequential groups). The antigens were adsorbed to aluminum hydroxide (0.5% *w/v* final concentration of Al(OH)_3_). The vaccines were administered subcutaneously. For serotypes A and B, mice were vaccinated once with either the HC fragment or the toxoid and challenged three weeks later with 1000 MsLD_50_ of the homologous serotype of botulinum toxin. For serotype E, mice were immunized three times with either the HC fragment or the toxoid and challenged with 1000 MSLD_50_ of BoNT/E 10 days after the last immunization. Mice were monitored for survival for 10 days post-challenge.

### 2.4. Horse Immunization

Horses of mixed races were purchased from local Israeli vendors. The horse immunization regimen included three primary vaccinations with 10 mg of HC fragment adsorbed to aluminum hydroxide (0.5% *w/v* final concentration of Al(OH)_3_) with a one-month interval. Thereafter, the horses were immunized every three months with 20 mg of adjuvant-free HC fragment. The vaccines were administered subcutaneously into several sites between the shoulders and thigh. Horse vaccination with the toxoid was described previously [21]. The neutralizing antibody concentration was determined according to the European pharmacopoeia [22].

### 2.5. Horse IgG Subclass Analysis

The analysis included two plasma samples from each horse vaccinated either with the H_C_ fragment or the toxoid. First, the total horse IgG was determined in each sample as described [23]. Thereafter, each sample was serially diluted 2-fold in coating buffer (50 mM Na_2_CO_3_, pH 9.6) from 1000 to 15.6 ng/mL and adsorbed to 96-well polycarbonate plates in duplicate (50 µL per well, 4 °C overnight incubation). The plates were then washed (0.9% NaCl, 0.05% Tween 20) and blocked for 1 h at 37 °C with TSTA buffer (50 mM Tris, 0.9% NaCl, 0.05% Tween 20, 2% bovine serum albumin; 200 µL per well). After washing, the plates were incubated (1 h, 37 °C) with a detecting antibody specific to the determined subclass. For IgGa and IgGb, the detection antibodies were mouse anti-horse IgGa (diluted 1:1000) and mouse anti-horse IgGb (diluted 1:1000), respectively. The plates were washed and incubated (1 h, 37 °C) with an alkaline phosphatase-conjugated donkey anti-mouse antibody (diluted 1:1000). Finally, the plates were washed, *p*-nitrophenyl phosphate was added, and the absorbance at 405 nm was determined after 12 min. The total IgG concentration that yielded absorbance above a threshold value of 0.1 was determined as the IgG titer. For IgG(T), the detection antibody was horseradish-peroxidase (HRP)-conjugated goat anti-horse IgG(T) (diluted 1:2000) (BioRad). Following the washing steps, Sure Blue Reserve TMB 1-component was added. The reaction was stopped with 0.5 N H_2_SO_4_ after 12 min, and the absorbance at 450 nm was determined. For each sample, the total IgG concentration that yielded absorbance above a threshold value of 0.1 was determined as the IgG titer.

### 2.6. Competitive ELISA

The assay included mixing several concentrations of horse anti-toxoid antibody with HRP-conjugated horse anti-H_C_ fragment antibody (prepared using a Lighting Link HRP conjugation kit, Innova Bioscience) at a constant concentration and transferring the mixture into a 96-well plate coated with toxoid. Following incubation (37 °C, 1 h), unbound antibodies were washed, and the assay was developed with Sure Blue Reserve TMB 1-component for 15 min. After the addition of stop solution, the absorbance was determined at 450 nm. The absorbance results were normalized to the absorbance obtained in control wells in which naïve horse antibody at the lowest dilution used for the corresponding horse anti-toxoid antibody was incubated with HRP-conjugated horse anti-H_C_ fragment.

### 2.7. Postexposure Antitoxin Treatment in Intoxicated Guinea Pigs

First, the guinea pig (Charles River UK) intramuscular LD_50_ (GP IM LD_50_) was determined in 330–380 g Dunkin Hartley Guinea pigs. The assay included intramuscular injection (200 µL) of serially 1.5-fold dilutions of BoNTs to the back right leg of guinea pigs (for each BoNT, six groups of *n* = 5 per dose were injected). Survival was monitored for eight days, and the LD_50_ was determined using the Karber method [24]. The mouse IP LD_50_ (M IP LD_50_) to GP IM LD_50_ translation factors of the BoNT/A, BoNT/B, and BoNT/E preparations used were 4.0, 4.9, and 55.6, respectively.

To evaluate horse anti-H_C_ efficacy, guinea pigs that were exposed to 4 GP IM LD_50_ were treated postexposure with either SDA or TDA (*n* = 20) at a human-normalized antitoxin dose (215 IU/kg for BoNT/A and BoNT/B and 122 IU/kg for BoNT/E, [25]). For BoNT/A- and BoNT/B-exposed animals, the treatment was administered 10 h post-exposure, and for BoNT/E-exposed animals, the treatment was administered 4 h post-exposure. The antitoxin was administered intramuscularly to the back left leg at a total volume of 200 µL. Every experiment included a control group (*n* = 5) injected with PBS similarly.

### 2.8. Statistical Analysis

Calculation of the ED_50_ (effective dose protecting 50% of the mice) was carried out with GraphPad Prism 5 software (GraphPad software, San Diego, CA, USA), employing nonlinear regression analysis. Comparison of equine IgG subclass distribution between the plasma of horses vaccinated with H_C_ fragments and horses vaccinated with toxoids was conducted with a two-tailed t test. The GP IM LD_50_ was determined using the Karber method [24].

## 3. Results

### 3.1. Recombinant BoNT Subunits and Botulinum Toxoids Present Similar Potency in Mice

**Figure 1:**
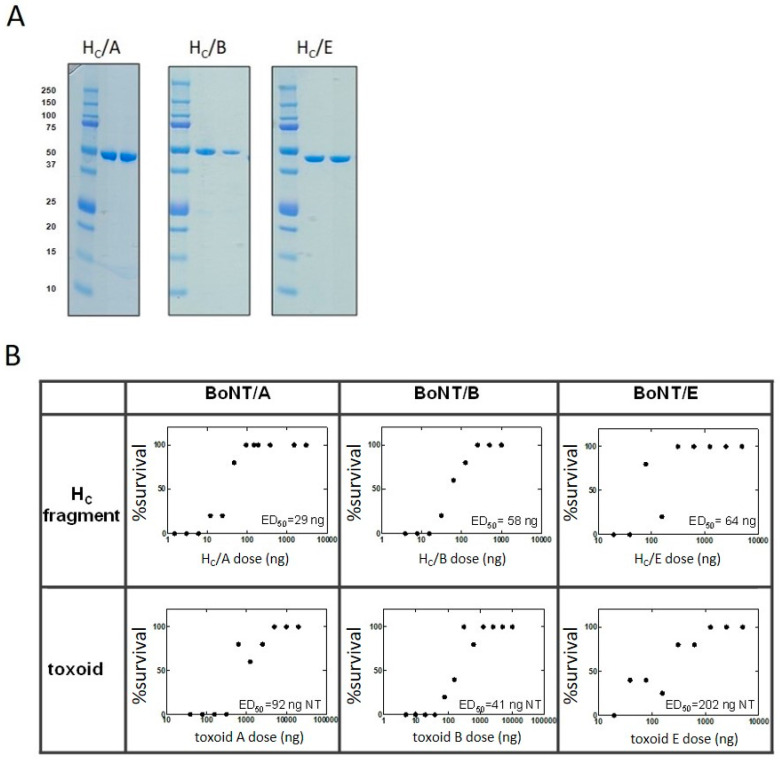
Comparative potency of recombinant BoNT subunits and botulinum toxoids in mice. (**A**) SDS-PAGE analysis of purified recombinant H_C_ fragments. The indicated H_C_ fragment serotype was separated in each corresponding gel under reducing (middle lane) or nonreducing (right lane) conditions. The left lane contains molecular weight marker with the indicated weight in kDa. (**B**) Nine groups of mice (5 per group) were vaccinated with serial doses (3.9–1000 ng) of either recombinant H_C_ subunits (upper panels) or botulinum toxoids (lower panels). For serotypes A and B, mice were vaccinated once, and for serotype E, mice received three vaccinations at one-month intervals. Three weeks after immunization with serotype A or B antigens and 10 days after the last vaccination with serotype E antigens, all mice were challenged IP with 1000 Ms IP LD50 of the homologous serotype of botulinum toxin. Mice were monitored for survival for 10 days, and the cumulative results were analyzed by nonlinear regression to calculate the ED50, which is the antigen dose that conferred 50% protection in mice. ED50 values are indicated within each graph for every separate BoNT subunit or toxoid.

Botulinum serotypes A, B, and E are responsible for >99% of clinical cases of human botulism. Equine antitoxin is the only approved anti-botulinum drug for adults. It is prepared by hyperimmunization of horses with a formaldehyde-inactivated form of the toxin (toxoid). The main objective of the current study was to test whether recombinant H_C_ fragments of BoNT/A, BoNT/B, and BoNT/E (recombinant BoNT subunits) could serve to generate a potent equine anti-botulinum antitoxin with therapeutic properties comparable to those of TDA antitoxin. The H_C_ fragments of BoNT/A, BoNT/B, and BoNT/E were expressed in *E. coli* and purified as previously reported [15,16], with high yields that can support horse immunization (hundreds of milligrams per liter of culture for H_C_/A and H_C_/E and dozens of milligrams of H_C_/B per liter of culture) (Figure 1A). The potency of recombinant BoNT subunits was initially evaluated in comparison to botulinum toxoids in mice (Figure 1B). Mice were immunized with increasing doses of H_C_ subunits or toxoids of botulinum serotypes A, B, or E. Three weeks (serotypes A and B-single immunization) or 10 days (serotypes E-triple immunization, 1-month intervals) after immunization, all mice were challenged intraperitoneally (IP) with 1000 Ms IP LD_50_ of the homologous serotype of botulinum toxin. Both H_C_/E and toxoid E were tested for potency after three vaccinations, whereas serotype A and B antigens were challenged after a single immunization. The reason for the difference in the potency protocols stems from the reduced immunogenicity of type E botulinum antigens [6,17].

The ED_50_ values for H_C_/A, H_C_/B, and H_C_/E were 29 ng, 58 ng, and 64 ng, respectively. These values coincide with those reported for H_C_ fragments expressed in *P. pastoris* and other expression systems [12,14,26]. The ED_50_ values of botulinum toxoids A, B, and E (92 ng, 41 ng, and 202 ng, respectively) were in the same range as those of the recombinant subunits, with a slightly increased ED_50_ for botulinum E toxoid. These results suggest that BoNT H_C_ fragments are at least as potent as botulinum toxoids in generating protection against botulinum intoxication and that they have the potential to generate potent equine anti-botulinum antitoxins.

### 3.2. A Single Boost with a High Dose of H_C_/E Significantly Elevates the Neutralizing Antibody Concentration (NAC) in a Toxoid-Immunized Horse

**Figure 2:**
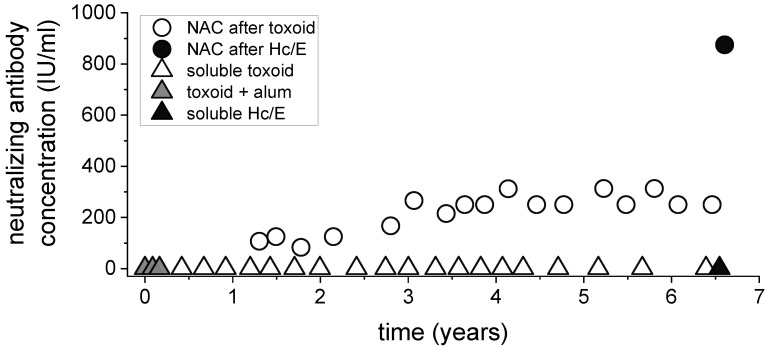
A boost with a high dose of recombinant H_C_/E dramatically elevates the NAC in a toxoid-immunized horse. Immunization schedule (triangles) and NAC (circles) in a horse immunized with 2 milligrams of type E botulinum toxoid over a period of 7 years. The first three immunizations (gray triangles) consisted of toxoid adsorbed to alum hydroxide, whereas all subsequent injections (white triangles) were with soluble toxoid. The NAC was determined in plasma collected three weeks after each boost. At the indicated time point, the horse was injected SC with a single elevated dose (40 mg) of recombinant Hc/E (black triangle). The neutralizing titer was measured in the plasma three weeks later and is indicated by a black circle.

Immunization of horses to produce hyperimmune antitoxin is a long process that demands substantial resources. To gain preliminary insight into the potential of BoNT subunits to elicit neutralizing antibodies in horses, the effect of a single boost with BoNT H_C_ was evaluated in a toxoid-hyperimmunized horse. A horse that was vaccinated with 2 mg of type E botulinum toxoid over a period of 7 years was selected for this purpose (Figure 2). Two years after the primary immunization with the toxoid, the NAC stabilized at a level of 200–250 IU/mL. This NAC meets the acceptance criteria to be used for the production of pharmaceutical F(ab’)_2_ antitoxin preparations as designated by the quality assurance at the IIBR cGMP production facility. One means to increase the neutralizing antibody concentration is by using elevated antigen doses. However, increasing the toxoid dose is challenging due to yield and safety limitations in obtaining a high concentration of active toxin and due to the limited yield of the detoxification process itself. As recombinant BoNT subunits are completely nontoxic and are produced at high yields, we tested how a booster injection with a high dose (40 mg) of Hc/E affected the NAC in this toxoid long-immunized horse (Figure 2). The single booster with a high dose of recombinant H_C_/E induced an over three-fold increase in the NAC from 250 IU/mL to 850 IU/mL. These results confirm that BoNT subunits are potent in inducing neutralizing antibodies in horses and that, due to their lack of safety and dose limitations, they may be used to further increase the NAC of toxoid-immunized horses.

### 3.3. Preparation and Characterization of BoNT Subunit-Derived Equine Antitoxins

**Figure 3:**
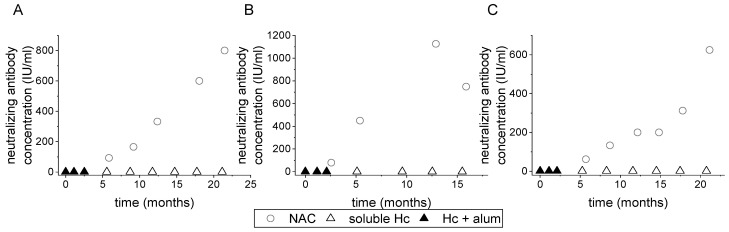
Immunization of horses with recombinant botulinum H_C_ subunits. Graphs present the immunization schedule (triangles) and neutralization titer (circles) in horses immunized with recombinant H_C_/A (**A**), H_C_/B (**B**), and H_C_/E (**C**). The first three immunizations (black triangles) consisted of 10 mg of recombinant H_C_ fragment adsorbed to alum hydroxide, whereas all subsequent injections (white triangles) contained 20 mg soluble Hc fragments.

The elevated NAC that was obtained after boosting a toxoid-immunized horse with a recombinant BoNT subunit vaccine served as a proof of concept for the utilization of these recombinant antigens for the production of equine botulinum antitoxins. To this end, three horses were individually immunized with recombinant H_C_/A, H_C_/B, or H_C_/E. The first three immunizations consisted of 10 mg recombinant H_C_ in aluminum hydroxide, whereas all the following injections were with 20 mg soluble Hc (Figure 3). An elevation in plasma NAC was observed as early as after the fourth immunization for all three recombinant H_C_ fragments (less than one year from the first injection). Within 24 months, plasma neutralization values reached 800 IU/mL in both H_C_/A- and H_C_/B-immunized horses and 600 IU/mL in the H_C_/E-immunized horse (Figure 3). The NACs obtained in H_C_-immunized horses were comparable to those of horses vaccinated with the corresponding toxoids. In addition, elevation in the NAC in the H_C_-immunized horses preceded that observed in toxoid-immunized horses.

These results indicate that H_C_ fragments are at least as potent as botulinum toxoids, suggesting that they can serve as a source of pharmaceutical-grade immunogens to induce neutralizing antitoxins for human use.

**Figure 4:**
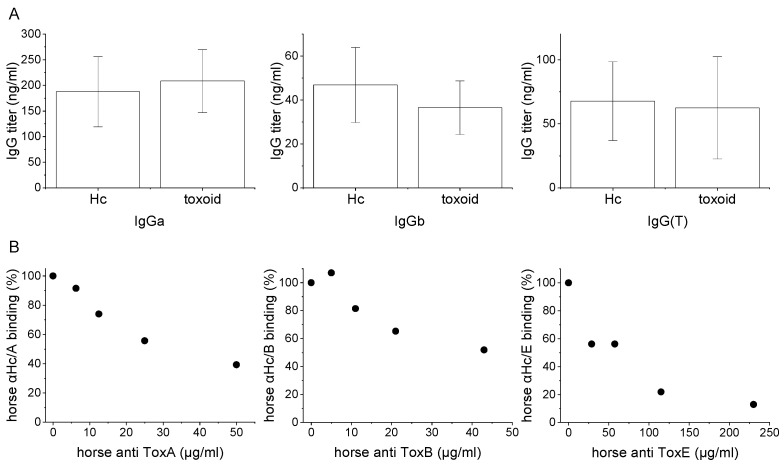
Characterization of subunit-derived antitoxin. (**A**) IgG subclass levels in the plasma of Hc- and toxoid-immunized horses. Plasma samples were serially diluted from 1000 to 15.6 ng/mL of total IgG, adsorbed to 96-well plates, and detected by a subclass-specific antibody as indicated. The lowest IgG concentration that induced a signal in each sample is indicated. The results are the average ± STD for each indicated group (Hc/A, H_C_/B, and H_C_/E or toxoid A, B, and E). The difference in IgG subclass frequency between Hc fragment-vaccinated horses and toxoid-vaccinated horses was not significant (*p* value of 0.52, 0.15 and 0.78 for IgGa, IgGb, and IgG(T), respectively). (**B**) Competitive binding of SDAs and TDAs. Serial dilutions of the indicated horse anti-toxoid IgG samples were incubated with a constant concentration of HRP-conjugated horse anti-Hc, and the mixtures were allowed to bind toxoid-coated plates. The absorbance results were normalized to the absorbance obtained in control wells in which naïve horse antibody at the lowest dilution used for the corresponding horse anti-toxoid antibody was incubated with HRP-conjugated horse anti-Hc fragment. For all BoNT serotypes, increasing the horse anti-toxoid IgG concentration resulted in reduced binding of horse anti-Hc, indicating that both preparations bind mutual epitopes on the toxoids.

The SDAs were subjected to comparative characterization to TDAs for their IgG subclass profiling and for binding their homologous toxins. Figure 4A depicts the distribution of IgG subclasses in the plasma of subunit- vs. toxoid-immunized horses. IgGa, IgGb, and IgG(T) levels were quantified and found to be similar with no statistically significant difference as analyzed by t-test (*p* = 0.52, 0.15 and 0.78 for IgGa, IgGb, and IgG(T), respectively, Figure 4A).

Next, a competitive ELISA was used to study whether SDA and TDA bind mutual epitopes on botulinum toxins. For that purpose, mixtures of serial dilutions of TDA were preincubated with a constant (A-8.4 µg/mL; B-8.4 µg/mL; E-14 µg/mL) concentration of HRP-conjugated SDA. The mixtures were then allowed to bind the homologous toxoid, and the HRP signal was measured. Of note, testing the opposite set-up, in which the SDA is titrated against a constant concentration of HRP-labeled-TDA, is impractical since antibodies from TDA also recognize epitopes on the additional domains of BoNTs (LC and H_N_). The binding of such antibodies to BoNTs will not be affected by the presence of SDA antibodies, causing a high background and not allowing us to observe subtle changes in the signal. All three serotype-specific TDAs inhibited SDA binding to the toxoid in a dose-dependent manner (Figure 4B). SDA binding was completely unaffected by naïve horse plasma at a concentration equivalent to the highest concentration of TDA tested. Hence, in addition to the similar profile of IgG subclasses in SDA and TDA, these preparations bind mutual epitopes on the corresponding toxoids.

### 3.4. Subunit-Derived Equine Antitoxin Is Fully Protective When Administered to Botulinum-Intoxicated Guinea Pigs

In the absence of preventive treatment and due to safety concerns in the systemic administration of equine-derived foreign proteins, botulinum antitoxins are labeled for use in humans only after exposure to botulinum toxins. To test the efficacy of equine SDA in a clinically relevant context, a human-calibrated dose of SDA or TDA against BoNT/A, BoNT/B, or BoNT/E was administered to guinea pigs postexposure to a lethal dose of BoNTs. Guinea pigs were injected IM with 4 GPLD_50_ of serotypes A, B, or E of botulinum toxin. Ten (serotypes A and B) or four (serotype E) hours post-intoxication, animals were treated IM by injection of 215 IU/kg subunit-derived or toxoid-derived (*n* = 10, each) anti BoNT/A or BoNT/B or 122 IU/kg of anti BoNT/E, to the opposite leg. Animals intoxicated with type E botulinum were treated earlier than those exposed to type A and type B botulinum in agreement with the differences in intoxication kinetics of these serotypes in humans and in animal models of botulism [25,27,28,29]. For each serotype, a control group (*n* = 5) was left untreated. SDA fully protected all intoxicated animals from the lethal dose of botulinum A, B, and E (Table 1). The efficacy of the SDA was identical to that of the TDA, suggesting that recombinant BoNT subunits may replace botulinum toxoids as antigens for the preparation of pharmaceutical anti-botulinum equine antitoxins.

## 4. Discussion

Botulinum serotypes A, B, and E are responsible for >99% of human botulism [30]. The current approved drug treatment consists mostly of equine antitoxin. In the United States, a human-derived antitoxin (BabyBIG) collected from vaccinated volunteers is available for infants [31]. Production of equine antitoxin relies on vaccination of horses with botulinum toxoid, which in turn involves laborious efforts under strict safety conditions for its preparation. In the current study, we aimed to compare the current TDA to a second-generation equine antitoxin derived from horses immunized with the recombinant receptor-binding domain of botulinum neurotoxin (H_C_ fragment). The recombinant subunits of botulinum serotypes A, B, and E were expressed in *E. coli* and were initially evaluated for their potency in mice compared to the corresponding botulinum toxoids. The potency of H_C_ fragments expressed both in eukaryotic and prokaryotic expression systems was previously reported. The ED_50_ of H_C_/A expressed in the methylotrophic yeast *Pichia pastoris* was reported to be in the range of 50–100 ng when tested in mice against a 1000 MsLD_50_ challenge. In our study, a similar value of 29 ng was determined in mice challenged with a similar dose of 1000 MsLD_50_. In contrast to H_C_/A, only a few studies have reported the immunogenic and protective properties of H_C_/B and H_C_/E. The potency of H_C_/B expressed in *Pichia pastoris* was in the same range as that expressed in *E. coli* (ED_50_ of 156 ng and 58 ng, respectively [16,26]). A more pronounced difference was found for H_C_/E expressed in the same expression systems, with ED_50_ values of 214 ng and 64 ng in *Pichia* and in the current study, respectively [32]. It should be noted yet that comparison between the ED_50_ values obtained for H_C_/E in these two particular studies is limited by differences both in the vaccination regimen and the toxin challenge dose.

Most importantly, we found the potency of the H_C_ fragments to be similar and in the same range as that of the toxoids. 

A single boost with H_C_/E induced a significant >3-fold increase in neutralizing antibody concentration from 250 IU/mL to 850 IU/mL in a horse vaccinated for over 7 years with botulinum type E toxoid. The H_C_/E boost dose (40 mg) was indeed 20-fold higher than the toxoid dose (2 mg) used for multiple immunizations of the horse. However, a significant increase in the toxoid dose is impractical due to limitations in toxin yield in *Clostridium botulinum* culture, toxoid preparation and safety concerns of these processes. During the seven years of immunization with toxoid, attempts to increase the titer by increasing the intervals between injections were not successful. Moreover, additional horses that were vaccinated using the same toxoid-base immunization protocol reached a similar titer range of 250–300 IU/mL. The 850 IU/mL titer obtained by the single high-dose booster with the recombinant H_C_/E fragment supports a previous study conducted in rabbits with increased-dose boosting [15] and confirmed that H_C_ fragments are suitable for horse immunization for the purpose of botulinum antitoxin preparations.

Horse-derived proteins are a major safety concern in the clinic when using equine antitoxins for snake bites or other biological intoxications [33]. The elevated NAC observed in H_C_-immunized horse plasma may have an important safety benefit, as the increased specific activity of such antitoxin results in reduced immunogenic horse-derived proteins per treatment dose compared to toxoid-immunized horse plasma.

Immunization of horses using a combined protocol of toxoid and H_C_ fragment is highly challenging in terms of quality assurance of a GMP product. Moreover, the relative contribution of H_C_-based antitoxin can only be evaluated by testing an immunization protocol that consists solely of H_C_ fragment as an antigen in naïve horses. For that purpose, horses were immunized with either H_C_/A, H_C_/B, or H_C_/E. All horses reached a NAC higher than 600 IU/mL within 16–20 months from the start of immunization. Titer started to increase earlier and after fewer injections when compared to the toxoid immunization protocol (demonstrated for toxoid E and H_C_/E in Figure 2 and Figure 3, respectively). Most importantly, the neutralizing antibody concentration obtained in all H_C_ fragment-immunized horses was comparable to that measured in toxoid-immunized horses in our facility and by others [34]. Analysis of equine IgG subclasses demonstrated that immunization with H_C_ fragments does not induce divergence from the antibody subclass profile developed in toxoid-vaccinated horses. In vitro characterization showed that all serotype-specific SDAs bind their corresponding counter toxoid in a dose-dependent manner. Importantly, TDAs inhibited SDA binding to the toxin, confirming that H_C_ fragment-related antitoxins bound the same relevant epitopes on the toxin (Figure 4).

Botulinum antitoxin is labeled for clinical use in humans only postexposure to the toxin. To evaluate the efficacy of SDA in a clinically relevant scenario, guinea pigs were exposed to a lethal dose of Type A, B, or E botulinum toxin and treated with the corresponding H_C_ fragment-related or toxoid-related antitoxins postintoxication. SDAs fully protected intoxicated guinea pigs and were at least as potent as their TDAs in their efficacy. Altogether, our results confirm that recombinant H_C_ fragments of botulinum toxins are suitable antigens for the preparation of equine antitoxins and that they can replace the laborious efforts needed to be conducted under stringent safety conditions to prepare toxoid antigens.

The results of the current study are supported by several previous works. Stahl et al. [35] immunized horses with recombinant H_C_/C and H_C_/D as a veterinary vaccine against botulinum serotypes that are endemic in horses. Although the dose of antigen used for immunization was significantly lower than that required to establish a hyperimmune state for the purpose of antitoxin preparation for passive immunization, the authors reported that H_C_ fragment-based vaccines elicited protective antibody titers similar or superior to the commercially available toxoid vaccine [35]. Yu et al. reported an experimental botulinum A equine antitoxin prepared by immunization of horses with H_C_/A [36]. The authors reported a peak NAC of 8000 IU/mL in horse sera after six immunizations with a mg-range H_C_/A, a value that is one order of magnitude higher than the titer established in the current study for H_C_/A. In both studies, H_C_/A was expressed in *E. coli*. The difference in neutralizing titer between the two studies could stem from differences in the potency assay methodology as instructed by the Chines and the European Pharmacopeia used in Yu et al. [36] and in the current study, respectively.

Switching from toxoid to recombinant H_C_ fragment as an immunogen for antitoxin production was reported in human-based antitoxin as well. The human antitoxin for infant botulism (BabyBig) is produced from volunteers who were immunized with a pentavalent-toxoid-derived vaccine for many years. In recent years, the antigen used for boost injections was switched to an H_C_-derived vaccine [10].

In conclusion, the data presented in the current study serve as a proof of concept for transferring the production of trivalent anti-botulinum A, B, and E equine antitoxins from a toxoid-based to a safer and more economical H_C_ fragment-based GMP product with a similar high efficacy.

## Figures and Tables

**Table 1:** Postexposure efficacy of subunit- and toxoid-derived antitoxin preparations in guinea pigs (survivals/number of animals in group).

	BoNT/A	BoNT/B	BoNT/E
Control	0/5	0/5	0/5
Subunit-derived antitoxin	20/20	20/20	20/20
Toxoid-derived antitoxin	19/20	20/20	20/20

## Data Availability

The datasets generated for this study are available on request to the corresponding author.
